# Comparative pharmacokinetics and pharmacodynamics of the advanced Retinol-Binding Protein 4 antagonist in dog and cynomolgus monkey

**DOI:** 10.1371/journal.pone.0228291

**Published:** 2020-01-24

**Authors:** Boglarka Racz, Andras Varadi, Paul G. Pearson, Konstantin Petrukhin

**Affiliations:** 1 Department of Ophthalmology, Columbia University, New York, New York, Unites States of America; 2 Pearson Pharma Partners, Westlake Village, California, United States of America; Wayne State University, UNITED STATES

## Abstract

Accumulation of lipofuscin bisretinoids in the retina contributes to pathogenesis of macular degeneration. Retinol-Binding Protein 4 (RBP4) antagonists reduce serum retinol concentrations thus partially reducing retinol delivery to the retina which decreases bisretinoid synthesis. BPN-14136 is a novel RBP4 antagonist with good *in vitro* potency and selectivity and optimal rodent pharmacokinetic (PK) and pharmacodynamic (PD) characteristics. To select a non-rodent species for regulatory toxicology studies, we conducted PK and PD evaluation of BPN-14136 in dogs and non-human primates (NHP). PK properties were determined following oral and intravenous administration of BPN-14136 in beagle dogs and cynomolgus monkeys. Dynamics of plasma RBP4 reduction in response to compound administration was used as a PD marker. BPN-14136 exhibited favorable PK profile in both species. Dose-normalized exposure was significantly higher in NHP than in dog. Baseline concentrations of RBP4 were considerably lower in dog than in NHP, reflecting the atypical reliance of canids on non-RBP4 mechanisms of retinoid trafficking. Oral administration of BPN-14136 to NHP induced a strong 99% serum RBP4 reduction. Dynamics of RBP4 lowering in both species correlated with compound exposure. Despite adequate PK and PD characteristics of BPN-14136 in dog, reliance of canids on non-RBP4 mechanisms of retinoid trafficking precludes evaluation of on-target toxicities for RBP4 antagonists in this species. Strong RBP4 lowering combined with good PK attributes and high BPN-14136 exposure achieved in NHP, along with the biology of retinoid trafficking that is similar to that of humans, support the choice of NHP as a non-rodent safety species.

## Introduction

**Fig 1:**
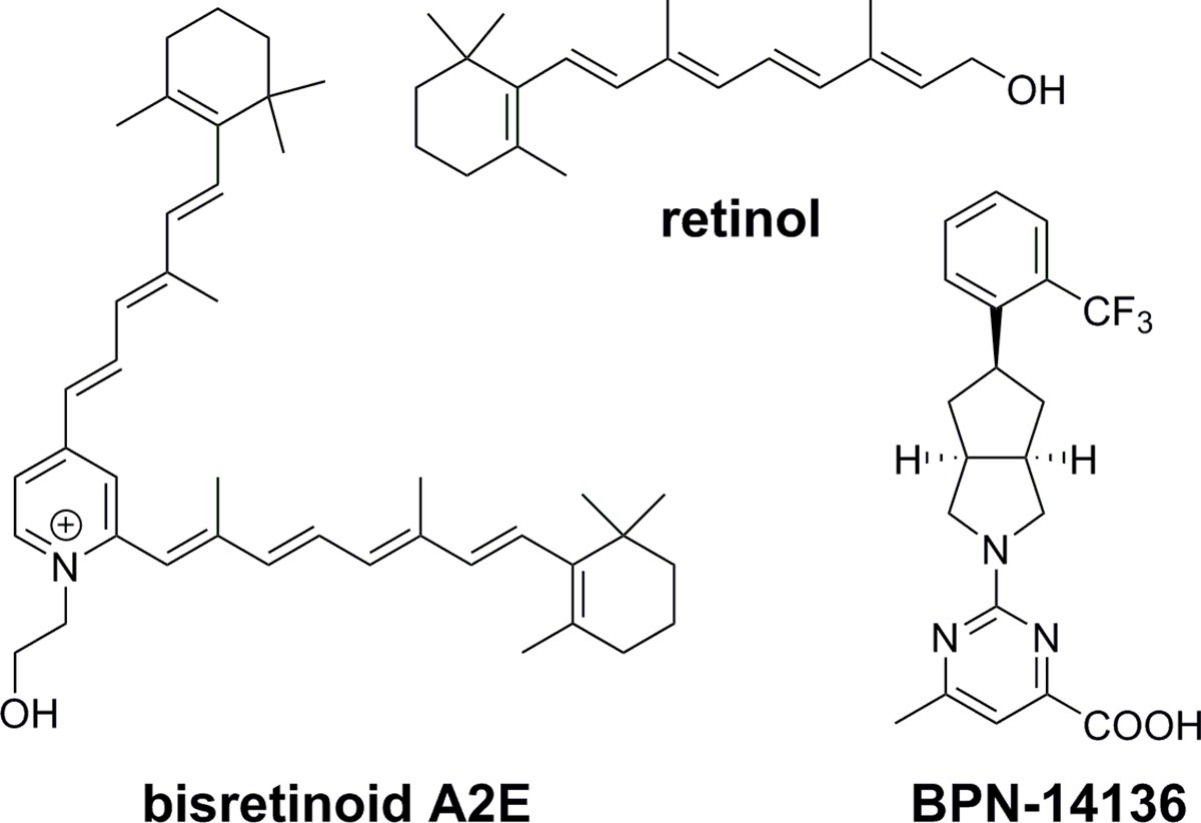
Chemical structure of RBP4 ligands retinol and BPN-14136 and bisretinoid N-retinylidene-N-retinylethanolamine (A2E).

Dry (atrophic) form of age-related macular degeneration (AMD) represents a slowly progressing neurodegenerative disorder in which specialized neurons (rod and cone photoreceptors) die in the central part of the retina called macula [1]. Photoreceptor loss in dry AMD is triggered by abnormalities in the retinal pigment epithelium (RPE) that provides critical metabolic support to these light-sensing neurons. Age-dependent accumulation of lipofuscin in the RPE matches the age-dependent increase in prevalence of dry AMD and thus is frequently considered as one of pathogenic factors contributing to the disease progression [2–8]. Enhanced accumulation of lipofuscin is believed to be the sole etiological factor in monogenic Stargardt disease, a genetic form of macular degeneration caused by mutations in the *ABCA4* gene [9]. Best Vitelliform Macular Dystrophy (BVMD) is another inherited form of early-onset macular degeneration characterized by abnormally high levels of retinal lipofuscin [10]. There are no FDA-approved treatments for dry AMD, Stargardt disease and BVMD. Given that lipofuscin toxicity is mediated by its bisretinoid components such as A2E (Fig 1), it was suggested that pharmacological inhibition of bisretinoid synthesis may delay or prevent photoreceptor loss in macular degeneration [11–15]. Bisretinoid synthesis occurs in the retina in a non-enzymatic manner from visual cycle retinoids such as all-*trans*- and 11-*cis*-retinaldehyde [16–18]. Uptake of retinol from circulation to the RPE fuels the visual retinoid cycle reactions leading to retinaldehyde synthesis and bisretinoid formation [1]. Given that bisretinoid synthesis depends on the influx of all-*trans*-retinol from serum to the RPE, it was suggested that pharmacological reduction of serum retinol may represent a target area in a search for macular degeneration treatment. Serum retinol is delivered to the RPE by the specific serum carrier protein, Retinol-Binding Protein 4 (RBP4) [19, 20]. Most of the retinol-RBP4 complex in circulation is bound with another serum protein, transthyretin (TTR) [20–22]. RBP4-TTR interaction increases the molecular weight of the retinol-delivery vehicle which is critical for maintaining serum retinol in circulation. Without complexation with TTR, RBP4-retinol is rapidly cleared from the bloodstream through glomerular filtration due to its small size, 21 kDa [23, 24]. Retinol binding to RBP4 is required for the formation of the RBP4-TTR complex, as apo-RBP4 has reduced affinity for TTR [21, 25]. We previously reported the discovery and characterization of several classes of nonretinoid RBP4 antagonists disrupting retinol-induced RBP4-TTR interaction [26–30]. One of the advanced analogs, BPN-14136 (Fig 1) has optimal drug-like characteristics and demonstrates very good *in vitro* RBP4 binding potency as well as a strong *in vitro* ability to antagonize retinol-dependent RBP4 interaction with TTR [27]. The compound showed good PK characteristics in rodents (mouse and rat) coupled with significant *in vivo* efficacy (plasma RBP4 lowering) in both rodent species [27, 28] which correlated with a desired partial reduction of retinaldehydes serving as direct bisretinoid precursors [28]. BPN-14136 dosing in the *Abca4*^*-/-*^ mouse model of Stargardt disease significantly inhibited bisretinoid synthesis and normalized dysregulation of the complement system in the retina [28]. To advance BPN-14136 characterization, we describe here an evaluation of its PK and PD properties in two non-rodent species, beagle dog and cynomolgus monkey, along with evaluation of additional relevant *in vitro* ADME (absorption, distribution, metabolism, and excretion) properties. The important objective of the PK-PD and *in vitro* ADME studies was the selection of the appropriate non-rodent species suitable for a formal evaluation of BPN-14136 safety in GLP studies as well as confirmation that canine retinal degeneration models, such as the *cmr* model of BVMD, can be used in accessing pre-clinical efficacy of BPN-14136 and similar compounds.

## Materials and methods

### BPN-14136 Synthesis and in vitro ADME tests

BPN-14136 was synthesized as described previously [27, 28]. *In vitro* ADME tests were conducted at AMRI, Albany, NY. Plasma protein binding for BPN-14136 was determined (in triplicates) by equilibrium dialysis of plasma against phosphate buffered saline (pH 7.4). Plasma spiked with BPN-14136 at a concentration of 1 μM was loaded to one side (donor) of the dialysis device insert, and phosphate buffered saline was loaded to the other side (receiver). After four hours, the concentration of BPN-14136 was assessed in both the donor and receiver sides.

Metabolic stability determinations for BPN-14136 and testosterone (positive control) were conducted in the presence of human, dog, and cynomolgus monkey liver microsomes. All measurements were done in duplicate. Pooled mixed gender human donor microsomes, male beagle dog microsomes and male cynomolgus monkey were obtained from BioIVT (Baltimore, MD). BPN-14136 was prepared as a 10 mM stock solution in DMSO. A mixture containing 50 mM potassium phosphate buffer pH 7.4 and 1 mg/mL liver microsomes was pre-warmed for 10 min at 37°C in a shaking water bath, followed by the addition of test compound. Final DMSO concentration in the reaction mix was 0.1%. Reactions with cofactor were initiated by adding a NADPH-regenerating system to the incubation mixtures (final concentrations of 1.3 mM NADP+, 3.3 mM glucose-6-phosphate, and 0.4 U/mL glucose-6-phosphate dehydrogenase). The final volume of the reaction mixture was 800 μL, containing 1 mg/mL liver microsomes, and 1 μM test compound. Aliquots (100 μL) of reaction mixtures were removed from the incubation plate at pre-defined timepoints and mixed with 150 μL of ice-cold acetonitrile, incubated on ice for 15 min, and samples were centrifuged (3,600 rpm, 10 min, 4°C) to precipitate protein. The supernatants were diluted 1:1 (v/v) with water containing the internal standard and subjected to the LC/MS analysis. The low limit of quantitation for BPN-14136 was0.01 μg/ml.

Metabolic clearance in hepatocytes experiments were conducted to assess BPN-14136 stability in the presence of human, dog, and cynomolgus monkey hepatocytes. All measurements were done in duplicate. Testosterone and 7-hydroxycoumarin were used as positive controls. Pooled cryopreserved human hepatocytes, cryopreserved male beagle dog hepatocytes, and cryopreserved male cynomolgus monkey hepatocytes were obtained from BioIVT (Baltimore, MD). Compound at 1 μM was incubated with 1 x 10^6^ cells. Final DMSO concentration in the reaction mix was 0.1%. Aliquots (100 μL) of reaction mixtures were removed from the incubation plate at different timepoints and mixed with 150 μL of ice-cold acetonitrile. After incubation on ice for 15 min, the samples were centrifuged (3,600 rpm, 10 min, 4°C) to precipitate protein. The supernatants were diluted 1:1 (v/v) with water containing internal standards and subjected to the LC/MS analysis. Hepatic clearance was calculated using the well-stirred liver model [31]

### Pharmacokinetic studies

Pharmacokinetic studies were conducted in male beagle dogs and male cynomolgus monkeys at SRI International, Menlo Park, CA. General procedures for animal care and housing were in accordance with the National Research Council (NRC) Guide for the Care and Use of Laboratory Animals (1996) and the Animal Welfare Standards incorporated in 9 CFR Part 3, 1991 and complied with guidelines set forth by the ARVO Animal Statement for the Use of Animals in Ophthalmic and Vision Research. During the course of the PK experiments, cynomolgus monkeys were individually housed in stainless steel primary enclosures in an AAALAC-accredited facility. Animals were fed a commercial monkey chow, and supplemental food was provided (fresh fruits, vegetables, seeds, and nuts). Reverse osmosis-purified drinking water was available at all times. Cynomolgus monkeys were maintained on a 12:12 hour light:dark cycle at the 64–84°F temperature and 30–70% humidity. Monkeys had access to enrichment opportunities (grooming devices, foraging devices, activity panels and mirrors) and were provided auditory stimuli and human interaction enrichment throughout the study. Blood samples were collected by venipuncture of a peripheral vein. Animals were acclimated to the squeeze back mechanism in their home cage by repeated, short periods of restraint during general husbandry procedures for at least 2 weeks prior to dosing. Animal were not removed from their cages for collection, and no anesthesia/analgesic agents were used for blood collection. Monkeys were observed at each study blood collection time point and twice daily at least 6 hours apart (a.m. and p.m.). None of the cynomolgus monkeys became ill during the course of the study and all primates returned to the home colony after the completion of the study. For PK experiments, beagle dogs were individually housed in enclosed run (≥ 4 ft x 6 ft). Dogs were exposed to their daily ration of food, Harlan Teklad 2025C Certified Global 25% Protein Dog Diet. Water was provided from the public water supply (municipal tap water) *ad libitum*. Dogs were maintained on a 12:12 hour light:dark cycle at the 72–73°F temperature and 40–71% humidity. Every effort was made to minimize, if not eliminate, pain and suffering in all animals in this study. No restraining devices were used in blood collecting procedures. Dogs were gentle hand-held to restrain by trained technicians with the dogs foreleg extended for sample collection of blood from cephalic vein. Animals were awake during blood sample collection with no anesthesia/analgesic agents used, and positive reinforcement by the technician was provided following collection. No animals were required to be euthanized during this study due to their health status. All dogs were released back to the colony after the study was completed. Male beagle dogs and male cynomolgus monkey received a single i.v. dose of a test compound at 0.5 mg kg^−1^ (dog) and 1 mg kg^−1^ (monkey) or a single p.o. dose of a test compound of at 2 mg kg^−1^ (dog) and 5 mg kg^−1^ (monkey). The number of animals that was used in PK experiments is 3 per study group. Intravenous administration vehicle was 3% DMA/45% PEG300/12% ethanol/40% sterile water; p.o. vehicle was 2% Tween 80 in 0.9% saline. Blood was collected from dogs and monkeys at pre-dose and at 5, 15 and 30 min, and 1, 2, 4, 6, 8, 12, 24, 36 and 48 h post-dose in the i.v. arms and at 15 and 30 min, and 1, 2, 4, 6, 8, 12, 24, 36 and 48 h post-dose in the p.o. arm. Blood samples were processed to plasma. Following protein extraction with acetonitrile, compound concentrations in plasma were measured by LC-MS/MS. The plasma drug level data were analyzed using WinNonlin software package; Model 201 (for i.v. bolus administration);was used for the analysis of i.v. data, and Model 200 (for extravascular administration) was used for the analysis of the p.o. data. Aliquots of pre-dose plasma samples, as well as aliquots of plasma PK samples, were used for the analysis of the plasmabiomarker, RBP4.

### Plasma RBP4 Measurements and Immunoblotting

Concentrations of RBP4 in cynomolgus monkey plasma samples (diluted 1:5000) were measured using the RBP4 (Human) ELISA kit (catalog number AG-45A-0035YTP-KI01, AdipoGen, Switzerland) following the manufacturer's instructions. RBP4 concentrations in dog plasma samples at the 1:3000 dilution were measured using the Canine RBP4 ELISA kit (catalog number NBP2-60464, Novus Biologicals, Centennial, CO) following the manufacturer's instructions.

For Western blotting, serum and plasma proteins were separated by NuPAGE Bis-Tris gels (4%–12%) (Novex, Life Technologies) and transferred to PVDF (Novex, Life Technologies). After blocking, membranes were incubated with rabbit polyclonal anti-human RBP4 antibodies (1:500, catalog number A0040, Dako) overnight at 4°C. Membranes were washed 3 times for 10 min in Tris-buffered saline (pH 7.5) containing 0.2% Tween (TBST), and then incubated in goat anti-rabbit IgG-HRP secondary antibody (1:1000; sc-2004; Santa Cruz Biotechnology Inc.) for 1 hour at room temperature. After washing, the protein bands were visualized with enhanced chemiluminescence labeling using the ECL immunoblotting detection system (catalog number 32106, ThermoFisher Scientific). The developed films were scanned and the pixel volumes of the bands were determined by using NIH’s ImageJ software, with the values in ratios of intensity. Transferrin loading control was detected using rabbit anti-human transferrin polyclonal antibodies (1:1000, catalog number ADI-VAP-EN004-D, Enzo). HEK293-expressed purified dog RBP4 (catalog number 70006-D08H, Sino Biological) and HEK293-expressed purified human RBP4 (catalog number 10354-H08H, Sino Biological) were used as control of antibody specificity in Western blot experiments.

## Results

As previously reported, BPN-14136 exhibits very good *in vitro* RBP4 binding potency (IC_50_ = 12.8 nM in SPA binding assay) as well as the superb *in vitro* ability to antagonize retinol-dependent RBP4 interaction with transthyretin (IC_50_ = 43.6 nM in HTRF RBP4-TTR interaction assay) [27]. The compound did not inhibit standard cytochrome (CYP) P450 enzymes (all CYP inhibition IC_50_ values > 34 μM), displayed very good stability in human and rat liver microsomes (>90% remaining at 30 min.), no PXR activation, and no significant off-target activity at the hERG channel or within a standard screening panel of fifty-five GPCRs, enzymes, ion channels, and transporters [27]. The compound showed a good pharmacokinetic (PK) profile in rat [27] and mouse [28] and normalized disease phenotype in the mouse model of Stargardt disease [28]. Encouraged by very good rodent PK characteristics and good pre-clinical efficacy in the Stargardt disease model, we conducted PK and PD studies of BPN-14136 in dogs and non-human primates and extended *in vitro* ADME characterization of this compound.

### In vitro ADME and pharmacokinetic (PK) characteristics of BPN-14136 in dog and cynomolgus monkey

In light of good *in vitro* ADME characteristics in rodents [27] we extended BPN-14136 characterization and performed similar *in vitro* ADME studies in dog and non-human primate. The pharmacological profile of BPN-14136 is presented in Table 1.

**Table 1:** *In vitro* ADME Profile for BPN-14136.

Microsomal Stability^a^			%PPB^b^			Hepatocyte CL_int_ ^c^ (μl/min/10^6^ cells)		
(% Remaining at 30 min)								
**Human**	**Dog**	**Cyno**	**Human**	**Dog**	**Cyno**	**Human**	**Dog**	**Cyno**
94	93	95	99.9 ±0.1	99.7 ±0.0	99.9 ±0.0	<0.7	<0.7	0.7

^a^Compound stability in liver microsomes

^*b*^%PPB, plasma protein binding (data are shown as average and standard deviation)

^*c*^Hepatocyte intrinsic clearance

BPN-14136 exhibited very good stability in human, dog and cynomolgus monkey liver microsomes. The observed CL_int_ values in hepatocytes suggest very low predicted hepatic clearance. The extent of plasma protein binding was in the high range for all three species with low fraction unbound. Satisfactory *in vitro* ADME characteristics justified BPN-14146 characterization in dog and non-human primate PK studies. Single dose PK studies were conducted with cynomolgus monkeys at 2 mg kg^−1^ i.v. and 5 mg kg^−1^ p.o. and with beagle dogs at 0.5 mg kg^−1^ i.v. and 2 mg kg^−1^ p.o. BPN-14136 possessed favorable PK profiles in cynomolgus monkey and dog (Table 2).

**Table 2:** *In vivo* PK data for BPN-14136 following i.v. and p.o. administration in beagle dog and cynomolgus monkey.

Species	Dose	CL^a^	C_max_^b^	T_max_^c^	T_1/2_^d^	V_ss_^e^ (mL/Kg)	AUC_last_^f^	AUC_inf_^g^	%F^h^
		(mL/h/kg)	(μg/mL)	(h)	(h)		(hr• μg/mL)	(hr• μg/mL)	
Cyno	1.0 mg kg^−1^ (i.v.)	5.25 ± 1.10	11.4 ± 0.6^i^	0.083	15.6 ± 3.3	113.0 ± 4.0	171.1 ± 27.0	196.2 ±42.2	84.3 ± 24.8
	5.0 mg kg^−1^ (p.o.)	NC	31.5 ± 0.2	1.67 ± 0.58	17.8 ± 4.2	NC	676.2 ± 128.9	826.6 ± 243.1	
Dog	0.5 mg kg^−1^ (i.v.)	47.0 ± 2.2	2.7 ± 0.1^i^	0.083	2.6 ± 0.2	622.0 ± 3.4	10.3 ± 0.5	10.7 ± 0.5	84.2 ± 10.4
	2.0 mg kg^−1^ (p.o.)	NC	7.5 ± 0.2	0.33 ± 0.14	2.9 ± 0.4	NC	35.4 ± 0.4	35.9 ± 4.4	

Dosing groups consisted of three male beagle dogs or male cynomolgus monkeys. Data represented as mean ± SD.

^*a*^Total body clearance.

^*b*^Maximum observed concentration of compound in plasma.

^*c*^Time of maximum observed concentration of compound in plasma. For the i.v. group, the first plasma collection time is listed as the T_max_.

^*d*^Apparent half-life of the terminal phase of elimination of compound from plasma.

^*e*^Volume of distribution at steady state.

^*f*^Area under the compound plasma concentration versus time curve from 0 to the last time point compound was quantifiable in plasma.

^*g*^Area under the compound plasma concentration versus time curve from 0 to infinity

^*h*^Bioavailability; F = (AUC_INFpo_ × Dose_iv_) ÷ AUC_INFiv_ × Dose_po_). NC, not calculated

^*i*^Maximum observed concentration at first time point

**Fig 2:**
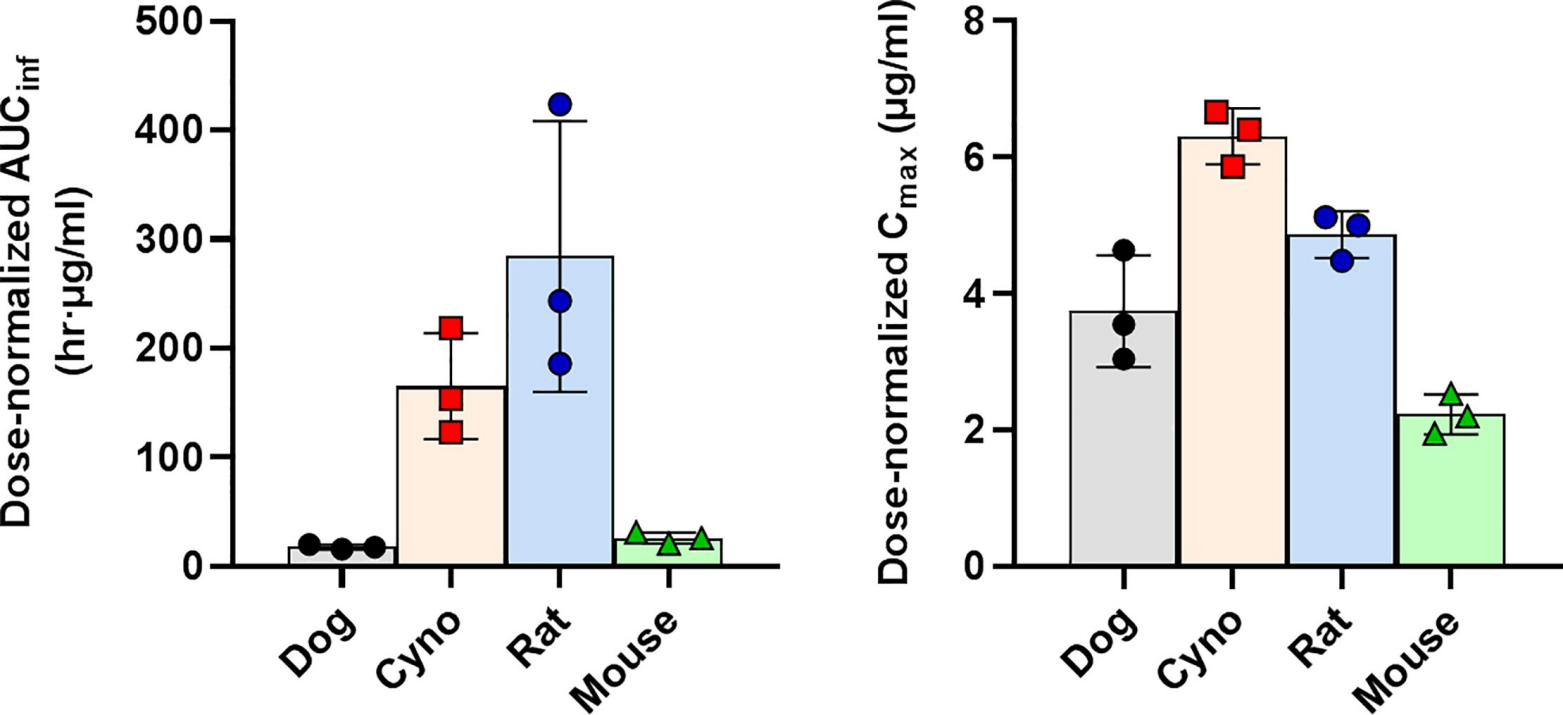
Comparison of dose-normalized exposure in four species after oral administration of BPN-14136. BPN-14136 was orally administered at the 5 mg kg^−1^ dose in cynomolgus monkey, rat and mouse and at the 2 mg kg^−1^ dose in dog. **A**, AUC_inf_, Area under the compound plasma concentration versus time curve from 0 to infinity normalized to the oral dose. **B**, C_max_, maximal plasma concentration of BPN14-136 normalized to the oral dose. Colored bars show data means; error bars show standard deviations. Each data point on the graph represents AUC_inf_ or C_max_ determined for an individual animal.

In non-human primates, BPN-14136 exhibited very low plasma clearance, with low V_ss_ values and a half-life of 17.8 h after oral dosing (Table 2). The compound was well absorbed and slowly eliminated from plasma after oral administration with an observed C_max_ of 31.5 μg/ml and corresponding T_max_ at 1.67 h. High exposures were observed (AUC_inf_ was 676.2 hr•μg/ml) and the estimated bioavailability was 84.3%. Comparably to cynomolgus monkey, BPN-14136 was rapidly absorbed in dog reaching the peak concentration of 7.5 μg/ml at 0.33 hr after post oral dose. CL and V_ss_ values were higher in dog than in cynomolgus monkey (Table 2). The observed plasma exposures (AUC_inf_) in dog ranged between 10.3 hr•μg/ml for i.v. dosing and 35.4 hr•μg/ml for oral administration. The oral bioavailabilities (%F) for BPN14136 for both species was around 84%. BPN-14136 exhibited overall good PK profile in both species (good oral bioavailability, moderate to low clearance and adequate exposure). The overall good PK profile of the compound in dog and NHP is consistent with good PK characteristics that BPN-14136 exhibited in our previous rat [27] and mouse [28] studies. Because the single oral dose administered in the dog PK study (2 mg kg^−1^) was lower than in PK experiments conducted in three other species (5 mg kg^−1^ in rat, mouse, and NHP) we compared exposure in four species by analyzing dose-normalized AUC_inf_ and C_max_ values (Fig 2). Dose-normalized AUC_inf_ and C_max_ values (AUC_inf_ /dose and C_max_/dose) were highest in cynomolgus monkey and rat while lower exposures were seen in mouse and dog PK studies (Fig 2). Given that high exposures may be needed to enable identification of target organ toxicity for BPN-14136 in future GLP toxicology studies, it seems reasonable to suggest that rat and NHP should be selected as rodent and non-rodent safety species, respectively.

### Steady-state concentrations of serum RBP4 in dog and cynomolgus monkey

**Fig 3:**
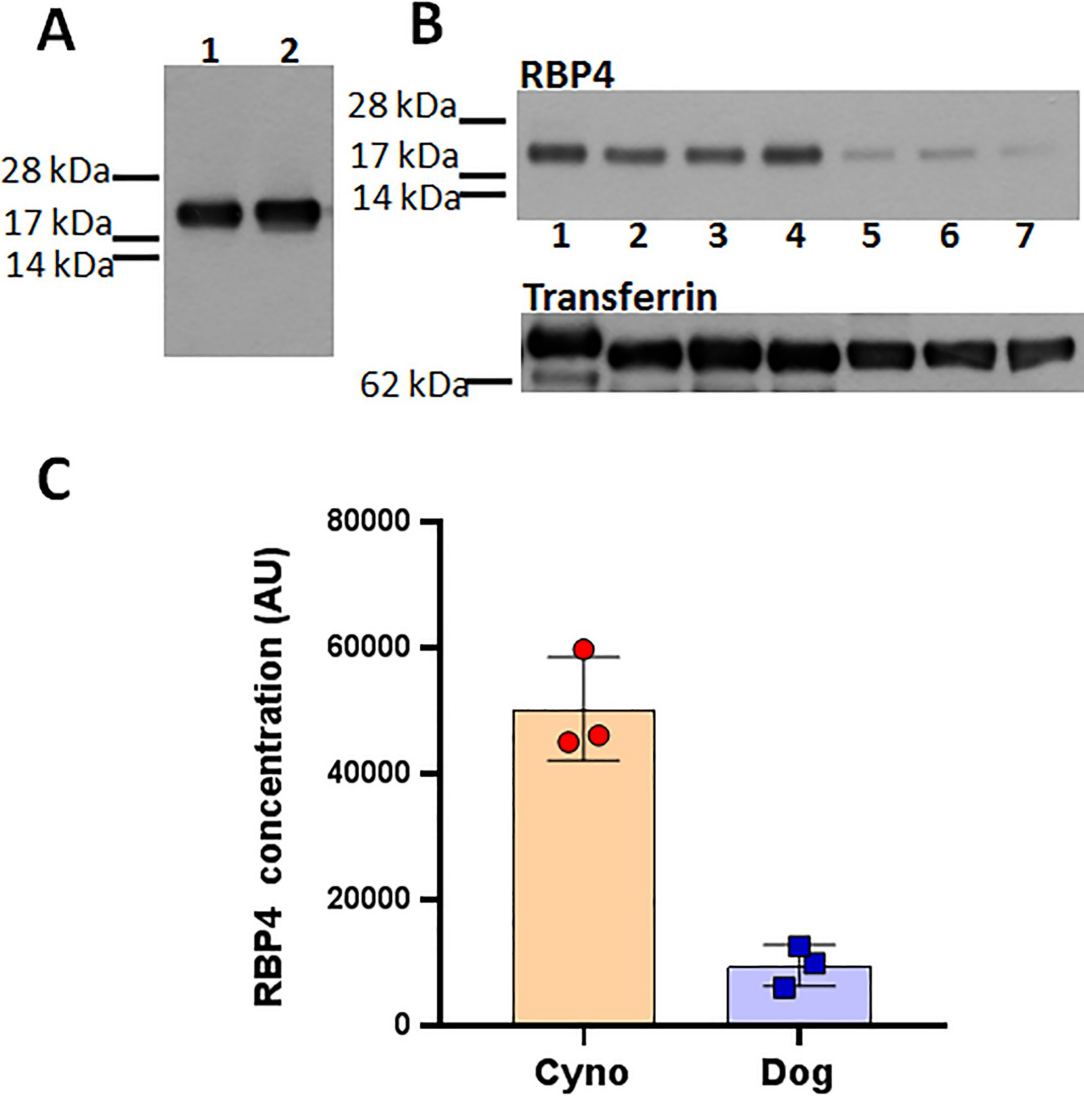
Western blots and a histogram showing steady-state levels of serum RBP4 in cynomolgus monkey and dog. **. A**, Western blotting confirms equal cross-reactivity of polyclonal rabbit anti-human RBP4 antibodies for dog and human RBP4. Samples (65 ng) of heterologously-expressed affinity-purified preparations of histidine-tagged dog (lane 1) and human (lane 2) RBP4 were probed with the antibodies. **B**, The representative immunoblot comparing RBP4 levels in 10 μl plasma samples from three dogs (lanes 2–4) and three cynomolgus monkeys (lanes 5–7). Human plasma sample (lane 1) was loaded as a reference control. Transferrin antibody was used to re-probe the Western blot to confirm equal loading of plasma samples (lower panel). **C**, The histogram representing pixel volumes of RBP4 bands. The bars represent pixel volume means ± S.D. of the scanned bands on the immunoblot in arbitrary units.

It has been previously reported that in contrast to other mammals, dogs and other carnivores deliver vitamin A to the target tissues predominantly as retinyl esters associated with lipoprotein fractions [32], while RBP4-TTR route of retinol delivery plays a secondary role. We wanted to assess whether this peculiarity of vitamin A trafficking in dogs may have an effect on pharmacodynamics of the RBP4 antagonist. As a first step, we compared steady-state RBP4 concentrations in dog and non-human primate plasma. Given that specific antibodies against canine RBP4 are not available, we performed Western blot analysis with rabbit polyclonal antibodies against human RBP4 (Dako, catlog number A0040). As shown in Fig 3A, this antibody demonstrated equal specificity for human and dog RBP4 when we probed a fixed amount of purified heterologously-expressed preparations of human and dog RBP4. Given that amino acid sequence of human RBP4 is 99% identical to the monkey orthologue, this immunoblot analysis of purified proteins confirmed that the antibodies are suitable for comparing concentrations of RBP4 in dog and NHP plasma. The analysis revealed a significant difference between dog and monkey plasma in concentration of RBP4 (Fig 3B). Consistent with the secondary role of the RBP4-TTR system for retinol delivery in dog, the canine plasma concentrations of RBP4 were approximately 5 times lower than those of monkey (Fig 3C).

### Pharmacodynamics (PD) of BPN-14136, and PK-PD correlations

**Fig 4:**
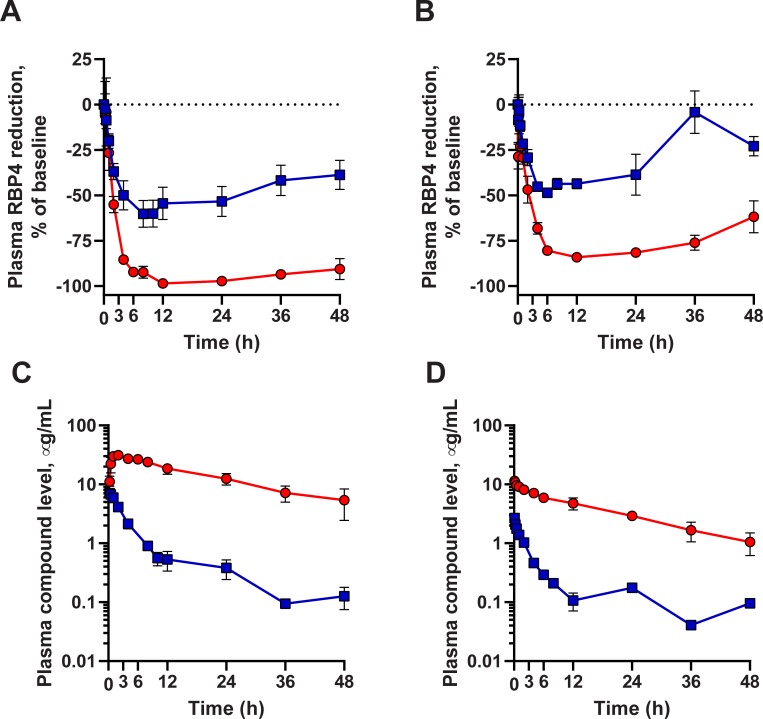
Pharmacodynamic and pharmacokinetic properties of BPN-14136 in cynomolgus monkey and beagle dog. Plasma RBP4 concentrations in monkeys and dogs following a single oral (*A*) and a single intravenous (*B*) administration of BPN-14136. (*C*, *D*) Plasma compound concentrations following administration of a single oral dose (*C*) and an intravenous dose (*D*) of BPN14136. Oral doses were 5 mg kg^−1^ in monkey and 2 mg kg^−1^ in dog experiments. Intravenous doses were 1 mg kg^−1^ in monkey and 0.5 mg kg^−1^ in dog experiments. Each dosing group consisted of three drug-naive adult males. Cynomolgus monkey graphs are plotted in red circles; beagle dog graphs are shown as blue squares. Each data point represents a mean of measurements from three animals ±SD.

Displacement of retinol from serum RBP4 by an RBP4 antagonist induces dissociation of the RBP4-TTR complex followed by renal clearance of RBP4 from circulation. Measurements of serum RBP4 concentrations represent a convenient pharmacodynamic marker for assessing the target engagement and *in vivo* potency of RBP4 antagonists. In order to compare *in vivo* RBP4-lowering efficacy of BPN-14136 in dog and cynomolgus monkey, we studied the effect of a single dose of the compound on concentrations of plasma RBP4. Aliquots of plasma samples collected during the PK experiments were used to analyze plasma RBP4 concentrations in the ELISA assay. After a single 5 mg kg^−1^ oral dose of BPN-14136 in NHP, a maximum of 99% decrease in plasma RBP4 was observed at 12 hours after dosing while maximal 60% plasma RBP4 reduction at 8 hours post-dosing was achieved following the 2 mg kg^−1^ oral administration of BPN-14136 in dogs (Fig 4A). The 1 mg kg^−1^ i.v. dose administered to monkey induced the 84% plasma RBP4 reduction while a lower 0.5 mg kg^−1^ i.v. dose used in dog yielded 50% plasma RBP4 lowering (Fig 4B). The dynamics of *in vivo* RBP4 lowering after oral and intravenous dosing of BPN-14136 in both species showed a general correlation between the presence of BPN-14136 in circulation (Fig 4*C* and 4*D*) and a reduction in serum RBP4 (Fig 4*A* and 4*B*). Very high C_max_, long exposure and slow clearance of BPN-14136 achieved after a single oral dose in cynomolgus monkey (Table 2) correlated very well with the magnitude and duration of the RBP4 lowering effect (97%, 94%, and 91% RBP4 reduction seen at the 24 h, 36 h, and 48 h time points, respectively). Consistent with lower C_max_, faster clearance and lower exposure achieved in dog PK experiments (Table 2), diminished RBP4-lowering effect was seen after oral and intravenous BPN-14136 administrations in beagle dogs (Fig 4A and 4B). However, appreciable concentrations of BPN-14136 within the 0.07–0.5 μg/mL range were detected in dog plasma at 24, 36, and 48 hours after oral dosing which correlated with the measurable reduction of RBP4 at these later time points (53%, 42% and 39% at 24 h, 36 h, and 48 h, respectively). Overall, our data provides evidence for a generally good PK-PD relationship between BPN-14136 exposure and biological response in both non-rodent species.

## Discussion

Biosynthesis of lipofuscin bisretinoids depends on the influx of serum retinol from circulation to the retina. Synthetic RBP4 ligands, such as BPN-14136, displace retinol from RBP4 and antagonize retinol-dependent RBP4-TTR interactions which induces rapid renal clearance of RBP4 diminishing retinol supply to the retina and causing inhibition of bisretinoid formation [33]. We previously established that BPN-14136 (Fig 1), which has very good *in vitro* RBP4 binding and RBP4-TTR antagonizing potency, was able to significantly reduce serum RBP4 in rat and mouse while showing good pharmacokinetic profiles in these two rodent species [27, 28]. Given that BPN-14136 significantly inhibited bisretinoid synthesis and normalized retinal complement system dysregulation in the mouse genetic model of Stargardt disease [28], this compound may be considered as a potential treatment candidate for dry AMD and other maculopathies. As the next step in advancing characterization of BPN-14136, we conducted PK-PD studies of this compound in two non-rodent species that could be selected for performing formal evaluation of BPN-14136 safety in GLP toxicology studies. Evaluation of relevant in vitro ADME properties such as metabolic stability in liver microsomes, hepatocyte clearance, and plasma protein binding (Table 1) did not reveal issues that could prevent conducting PK-PD studies of BPN-14136 in dogs and cynomolgus monkey. The PK study conducted in cynomolgus monkey established that BPN-14136 can be very well absorbed resulting in oral bioavailability of about 84% with very slow elimination, long half-life, high exposure (AUC_INF_ of 196.2 μg /ml∙hr), and an observed C_max_ of 31.4 μg/ml after oral administration and corresponding T_max_ at 1.67 h (Table 2). Bioavailability of BPN-14136 in dog, 84%, was similar to the one seen in the NHP study. However, BPN-14136 clearance in dog was faster than in cynomolgus monkey with maximal plasma concentration (C_max_) of 7.5 μg/ml achieved at T_max_ of 0.33 h post oral dose (Table 2). Given that a single oral dose administered in the dog PK study (2 mg kg^−1^) was lower than in PK experiments conducted in cynomolgus monkey as well as in two rodent species that we studied earlier (5 mg kg^−1^ in rat, mouse, and NHP) we compared exposure in four species by analyzing dose-normalized AUC_inf_ and C_max_ values (Fig 2). Dose-normalized exposure was significantly higher in cynomolgus monkey than in dog (Fig 2). As part of the safety assessment of new drugs, the use of two species (a rodent and a non-rodent) for regulatory toxicology studies is the typical approach taken for small molecules. To reveal all potential toxicities, these regulatory safety studies are conducted at doses that guarantee large exposure multiples over potential clinical exposures in humans. Based on the analysis of dose-normalized exposure of BPN-14136 in four species (Fig 2), it seems that cynomolgus monkey and rat may represent the optimal combination of non-rodent and rodent safety species in which highest exposure margins can be accomplished. One other criterion in selecting the appropriate species for non-clinical safety assessment is the presence of intended pharmacology. Serum retinol levels in humans and cynomolgus monkey are reported to be identical, and RBP4-TTR system seems to be the major route of retinoid delivery to vitamin A-dependent tissues [34]. In contrast, the majority of plasma vitamin A in dog (70%) is transported as retinyl esters associated with lipoproteins, and only 30% as retinol bound to RBP4 [32]. Predominant reliance on lipoprotein bound retinyl esters for delivery of vitamin A to target organs is a peculiarity of members of the canid family that clearly distinguishes them from other mammals [35]. Consistent with the reports that only 30% of serum vitamin A is transported in dog as RBP4-retinol, we determined that serum RBP4 concentrations in dog were significantly lower than that in cynomolgus monkey (Fig 3). Despite the lower steady state RBP4 concentrations in dog in comparison to monkey, the dynamics of plasma RBP4 concentrations in response to BPN-14136 administration was generally similar in both species. Significant 99% RBP4 reduction was achieved in cynomolgus monkey after administration of a single 5 mg kg^−1^ oral dose of BPN-14136 while 60% plasma RBP4 lowering was achieved in dog following administration of the lower 2 mg kg^−1^ oral dose (Fig 4). The magnitude and duration of *in vivo* RBP4 lowering after oral and intravenous dosing of BPN-14136 in dog and monkey seems to correlate well with the compound exposure in these two non-rodent species (Table 2 and Fig 4). Even though the PD response and PK characteristics in dog were satisfactory, this species may not be optimal for the assessment of on-target toxicities, given that dog predominantly relies on non-RBP4 mechanisms of vitamin A supply to the target organs. The reliance of dog on plasma lipoprotein-retinyl ester complexes for vitamin A delivery also indicates that canine models of retinal degeneration may not be used in assessing pre-clinical efficacy of RBP4 antagonists. We previously established very good efficacy of BPN-14136 in the mouse model of Stargardt disease [28], and it would be very interesting to assess this compound in relevant models of other genetic forms of macular degeneration, such as Best diseases. To date, the *cmr* dog model is the only reliable and well-studied model that mimics significant aspects of Best vitelliform macular degeneration [36]. However, BPN-14136 and other RBP4 antagonists may not be able to significantly inhibit bisretinoid synthesis in the canine model, given that retinoid delivery in dog is largely RBP4-independent.

A single oral dose of BPN-14136 in cynomolgus monkey induced a 99% reduction in serum concentrations of RBP4 (Fig 4), suggesting a complete pharmacological blockade of the RBP4-TTR-mediated retinol transport. Even though a lesser extent of RBP4 reduction may be required for clinical efficacy in dry AMD [37], this significant RBP4 lowering raises a concern on whether a highly potent RBP4 antagonist may induce clinically significant adverse effects due to systemic vitamin A deficiency. However, pharmacological suppression of serum RBP4 cannot be considered as similar to systemic vitamin A deprivation. While the RBP4-TTR complex is the primary transporter for retinol in the blood, retinoids are also supplied to vitamin A-dependent tissues through alternative, RBP4-indipendent routes [38–43]. Following absorption from the gut, dietary retinoids are packaged in chylomicrons, which are delivered primarily to the liver. However, 25–33% of retinol-laden chylomicrons are taken by extrahepatic tissues such as the retina [38]. Additionally, retinoic acid can also be transported to target organs in a complex with serum albumin [39]. *De novo* biosynthesis of vitamin A from dietary β-carotene in RPE cells and other tissues has also been reported [39, 44] highlighting yet another RBP4-independent route of retinoid supply. Patients without RBP4 in their blood due to compound heterozygous missense mutations in RBP4 display no clinical symptoms of systemic vitamin A deficiency [42]. Genetic ablation of *Rbp4* in mice does not lead to systemic abnormalities [38, 40, 41] or histological signs of retinal degeneration [45]. We recently reported that administration of BPN-14136 in mice at doses inducing 90% serum RBP4 lowering partially reduced the retinoid load in the retina and significantly inhibited bisretinoid synthesis without inhibiting the rate of the visual cycle or affecting retinal function as assessed by electroretinography [28]. It therefore seems unlikely that partial pharmacological reduction of serum RBP4 by RBP4 antagonists to precipitate symptoms of systemic vitamin A deficiency in individuals who maintain a standard vitamin A- and β-carotene-sufficient diet.

In summary, we report that despite adequate PK characteristics and good PD response of BPN-14136 in dog, this species is not optimal for conducting safety assessment of RBP4 antagonists due to the predominant reliance of canines on non-RBP4 mechanisms of retinoid trafficking which precludes evaluation of on-target toxicities for BPN-14136 and similar compounds. Significant RBP4 lowering and good PK characteristics with very high BPN-14136 exposure achieved in NHP, along with standard biology of retinoid trafficking, support the choice of NHP as a non-rodent safety species.

## Supporting information

S1 Table BPN-14136 plasma levels following a single oral or intravenous dose administration in dog and cynomolgus monkey. (PDF)

S1 File LC-MS/MS protocols and QC data for the analysis of dog and cynomolgus monkey plasma samples. (PDF)

S2 File The original and unadjusted western blot images. (PDF)
