# Supplementary material for: Comparative pharmacokinetics and pharmacodynamics of the advanced Retinol-Binding Protein 4 antagonist in dog and cynomolgus monkey
Source: PLoS One. 2020 Jan 24;15(1):e0228291. doi: 10.1371/journal.pone.0228291 (PMC6980506; doi:10.1371/journal.pone.0228291)
Supplement: S1 Table — (PDF) [file pone.0228291.s001.pdf]

Pharmacokinetics of BPN-14136 Following Single Intravenous or Oral Dose Administration to Non-Human Primates

Table 1

BPN-14136 Plasma Levels, Dose Route: iv, 1.0 mg/kg

	Time	Plasma (ng/ml)		
Animal #	(hr)	Individual	Mean	SD
1011-1008447	Predose	132		
1015-1002545	Predose	64.6		
1021-091131	Predose	392	196	173
1011-1008447	0.0083	10900		
1015-1002545	0.0083	11200		
1021-091131	0.0083	12100	11400	624
1011-1008447	0.25	10800		
1015-1002545	0.25	10500		
1021-091131	0.25	10900	10733	208
1011-1008447	0.5	9300		
1015-1002545	0.5	10000		
1021-091131	0.5	9400	9567	379
1011-1008447	1	9470		
1015-1002545	1	8350		
1021-091131	1	9370	9063	620
1011-1008447	2	8910		
1015-1002545	2	7340		
1021-091131	2	8000	8083	788
1011-1008447	4	7450		
1015-1002545	4	6800		
1021-091131	4	7000	7083	333
1011-1008447	8	6160		
1015-1002545	8	5290		
1021-091131	8	6220	5890	520
1011-1008447	12	4470		
1015-1002545	12	3860		
1021-091131	12	6000	4777	1102
1011-1008447	24	2840		
1015-1002545	24	2510		
1021-091131	24	3430	2927	466
1011-1008447	36	1610		
1015-1002545	36	1090		
1021-091131	36	2300	1667	607
1011-1008447	48	962		
1015-1002545	48	671		
1021-091131	48	1530	1054	437

Plasma Lower Limit of Quantitation (LLOQ) = 10.0 ng/ml

Pharmacokinetics of BPN-14136 Following Single Intravenous or Oral Dose Administration to Non-Human Primates

Table 2

BPN-14136 Plasma Levels, Dose Route: po, 5 mg/kg

Animal #	Time (hr)	Plasma (ng/ml) Individual	Mean	SD
1011-1008447	Predose	< LLOQ		
1015-1002545	Predose	< LLOQ		
1021-091131	Predose	< LLOQ	< LLOQ	NA
1011-1008447	0.25	9720		
1015-1002545	0.25	9420		
1021-091131	0.25	14200	11113	2677
1011-1008447	0.5	28100		
1015-1002545	0.5	15200		
1021-091131	0.5	23400	22233	6529
1011-1008447	1	33300		
1015-1002545	1	24600		
1021-091131	1	31400	29767	4574
1011-1008447	2	32400		
1015-1002545	2	29300		
1021-091131	2	32000	31233	1686
1011-1008447	4	26600		
1015-1002545	4	25800		
1021-091131	4	29100	27167	1721
1011-1008447	6	26700		
1015-1002545	6	22400		
1021-091131	6	30600	26567	4102
1011-1008447	8	23900		
1015-1002545	8	21700		
1021-091131	8	26200	23933	2250
1011-1008447	12	16700		
1015-1002545	12	16100		
1021-091131	12	22900	18567	3765
1011-1008447	24	12700		
1015-1002545	24	9590		
1021-091131	24	15100	12463	2763
1011-1008447	36	6800		
1015-1002545	36	5230		
1021-091131	36	9530	7187	2176
1011-1008447	48	4550		
1015-1002545	48	2950		
1021-091131	48	8650	5383	2940

Plasma Lower Limit of Quantitation (LLOQ) = 10.0 ng/ml
NA: Not Applicable

Pharmacokinetic Study of BPN-14136 Following a Single Dose Administration to Male Beagle Dogs

Table 3
BPN-14136 Plasma Levels, Dose Route: iv, 0.5 mg/kg

Animal #	Time (hr)	Plasma (ng/ml) Individual	Mean	SD
1	0.083	2660		
2	0.083	2750		
3	0.083	2650	2687	55.1
1	0.25	2000		
2	0.25	2090		
3	0.25	2140	2077	70.9
1	0.5	1800		
2	0.5	1690		
3	0.5	1820	1770	70.0
1	1	1500		
2	1	1270		
3	1	1380	1383	115
1	2	1020		
2	2	1060		
3	2	1000	1027	30.6
1	4	442		
2	4	492		
3	4	458	464	25.5
1	6	308		
2	6	284		
3	6	281	291	14.8
1	8	217		
2	8	203		
3	8	207	209	7.21
1	12	69.2		
2	12	141		
3	12	111	107	36.1
1	24	169		
2	24	186		
3	24	171	175	9.29
1	36	33.3		
2	36	47.1		
3	36	42.5	41.0	7.03
1	48	92.8		
2	48	102		
3	48	93.0	95.9	5.25

Plasma Lower Limit of Quantitation (LLOQ) = 10.0 ng/ml

Pharmacokinetic Study of BPN-14136 Following a Single Dose Administration to Male Beagle Dogs

Table 4
BPN-14136 Plasma Levels, Dose Route: po, 2 mg/kg

	Time	Plasma (ng/ml)		
Animal #	(hr)	Individual	Mean	SD
4	0.25	7100		
5	0.25	9280		
6	0.25	4890	7090	2195
4	0.5	6340		
5	0.5	7730		
6	0.5	6080	6717	887
4	1	5100		
5	1	6630		
6	1	6080	5937	775
4	2	3600		
5	2	4460		
6	2	4300	4120	457
4	4	1790		
5	4	2560		
6	4	2080	2143	389
4	8	855		
5	8	971		
6	8	885	904	60.2
4	10	407		
5	10	581		
6	10	690	559	143
4	12	453		
5	12	391		
6	12	753	532	194
4	24	330		
5	24	542		
6	24	277	383	140
4	36	82.4		
5	36	95.3		
6	36	105	94.2	11.3
4	48	152		
5	48	160		
6	48	67.0	126	51.5

Plasma Lower Limit of Quantitation (LLOQ) = 10.0 ng/ml
