# Supplementary material for: Comparative pharmacokinetics and pharmacodynamics of the advanced Retinol-Binding Protein 4 antagonist in dog and cynomolgus monkey
Source: PLoS One. 2020 Jan 24;15(1):e0228291. doi: 10.1371/journal.pone.0228291 (PMC6980506; doi:10.1371/journal.pone.0228291)
Supplement: S1 File — (PDF) [file pone.0228291.s002.pdf]

Pharmacokinetics of BPN-14136 Following Single Intravenous or Oral Dose Administration to Non-Human Primates

II. MATERIALS AND METHODS

A. Materials

Acetonitrile and formic acid were purchased from Mallinckrodt Baker, Inc. (Phillipsburg, NJ). Dimethyl sulfoxide (DMSO) was purchased from Sigma-Aldrich (St. Louis, MO). Milli-Q water was obtained in-house. Cynomolgus monkey plasma was purchased from Bioreclamation Inc. (Hicksville, NY). The analytical HPLC column was purchased from Phenomenex Inc. (Torrance, CA).

BPN-14136 and BPN-13671 were provided by the sponsor and the purity was assumed to be 100%.

B. Bioanalytical Method

Calibration Standards and QCs. Cynomolgus monkey plasma calibration standards and quality control (QC) samples were prepared in untreated plasma as follows. A primary stock solution of 1 mg/ml analyte in DMSO was prepared, then dilutions performed to make spiking solutions of analyte in DMSO at various concentrations. One volume of spiking solution was added to 99 volumes of blank plasma to attain nominal concentrations of standards with a final non-plasma matrix concentration of 1.0%. The calibration standards and QCs were stored at -70°C until analysis, and duplicate aliquots of the standards used in the plasma sample analysis to generate the calibration curve, and n=4 replicates of the QCs per concentration used to monitor method accuracy.

The calibration standard and QC concentrations (ng/ml) in the plasma analyses were as follows: Standards: 10.0, 50.0, 100, 500, 1010, 4000, 7000 and 10000. QCs: 20.0, 4500, and 9500. Plasma blanks were also prepared and included in the sample analyses.

Plasma Extraction Method: Plasma standards/samples were stored frozen at -70°C and thawed to room temperature for approximately 20 minutes on the day of analysis. The extraction method was as follows: To 50 µl of plasma in a 2 ml microfuge tube was added 300 µl acetonitrile containing 500 ng/ml BPN-13671 (internal standard). Tubes were vortexed at maximum speed on a plate vortexer 5 minutes, centrifuged 8 minutes at approximately 18,000 g, and 40 µl of the supernatant removed to a glass

HPLC vial containing 1000 µl of 85/15/0.1: water/acetonitrile/formic acid (v/v/v). The vials were briefly vortexed to mix, prior to LC-MS/MS analysis.

LC-MS/MS Method: Samples were analyzed by LC-MS/MS using the following conditions:

LC Conditions

Instruments:	Shimadzu LC-20AD Pumps<br>Leap Technologies CTC HTS PAL Autosampler
Autosampler Temperature:	10°C
Column:	Phenomenex Luna C18(2) 50 x 2 mm, 5 µm
Column Temp.:	Room temperature
Mobile Phase:	A= 0.1% formic acid in water (v/v)<br>B= 0.1% formic acid in acetonitrile (v/v)
Elution Mode:	Gradient;

Time (min)	%A	%B	Flow rate (ml/min)
0.0	98	2	1.0
0.7	98	2	1.0
1.7	5	95	1.0
3.5	5	95	1.0
3.7	98	2	1.0
5.2	98	2	1.0

Compound	Retention Time (min)
BPN-14136	1.89
BPN-13671 (IS)	1.77

Injection Volume:	10 µl
Strong Needle Wash Solvent:	99/1: methanol/ammonium hydroxide (v/v)
Weak Needle Wash Solvent:	20/80/0.1: acetonitrile/water/formic acid (v/v/v)

MS Conditions

Instrument:	AB Sciex QTrap 5500
Ionization:	Electrospray ionization, positive ion mode
Detection:	Multiple Reaction Monitoring (MRM);

Analyte	MRM Transition (m/z)	Collision Energy (eV)	Declustering Potential
BPN-14136	392.2→346.2	33	70
BPN-13671 (IS)	389.2→159.1	60	110

Dwell Times:	75 msec
Resolution:	Unit mass resolution at Q1 and Q3
Curtain Gas:	32
Ion Spray Voltage:	4500V

Desolvation Temperature:	600° C
Ion Source Gas 1:	70
Ion Source Gas 2:	90
Entrance Potential:	6V (BPN-14136), 10V (BPN-13671)
Exit Potential:	18V (BPN-14136), 20V (BPN-13671)
Quantitation:	Integration and Quantitation by Analyst Software ver 1.4.2

Calibration Curves. Calibration standard curves were prepared as follows: the peak area of BPN-14136 was divided by the peak area of BPN-13671 (internal standard) to yield a peak area ratio (PAR). The resulting PARs vs. nominal concentrations for calibration standards were fitted to a $1/x^2$ weighted quadratic regression, to generate a calibration curve for quantitation of study samples.

These calculations were performed by the Analyst software. The goodness of fit of the standard curve is indicated by the coefficient of determination (r^2) obtained from the quadratic regression, with perfect fit yielding an r^2 value of 1.000.

Lower Limit of Quantitation (LLOQ). The LLOQ was defined as the lowest concentration that could be measured yielding back-calculated accuracy of at least 50% of the standards within 80-120% of nominal, and was set as the lowest concentration in the standard curve. In the plasma sample analyses the LLOQ was 10.0 ng/ml.

Linearity and Range: The range of the plasma standard calibration curve (LLOQ to upper limit of quantitation, ULOQ) was 10.0 to 10,000 ng/ml. The coefficient of determination (r^2) was ≥ 0.997 in the sample run. The calibration curve from the plasma sample run is shown in Figure B-1. For sample concentrations above the ULOQ, samples were analyzed after diluting with blank plasma, so that the measured concentration was within the linear range of the assay, then a correction factor applied to the resulting measured value.

Accuracy and Precision of QC Samples: The accuracy (% of nominal) and precision (%CV) of the QC samples analyzed in the plasma sample analyses are shown in Table B-1.

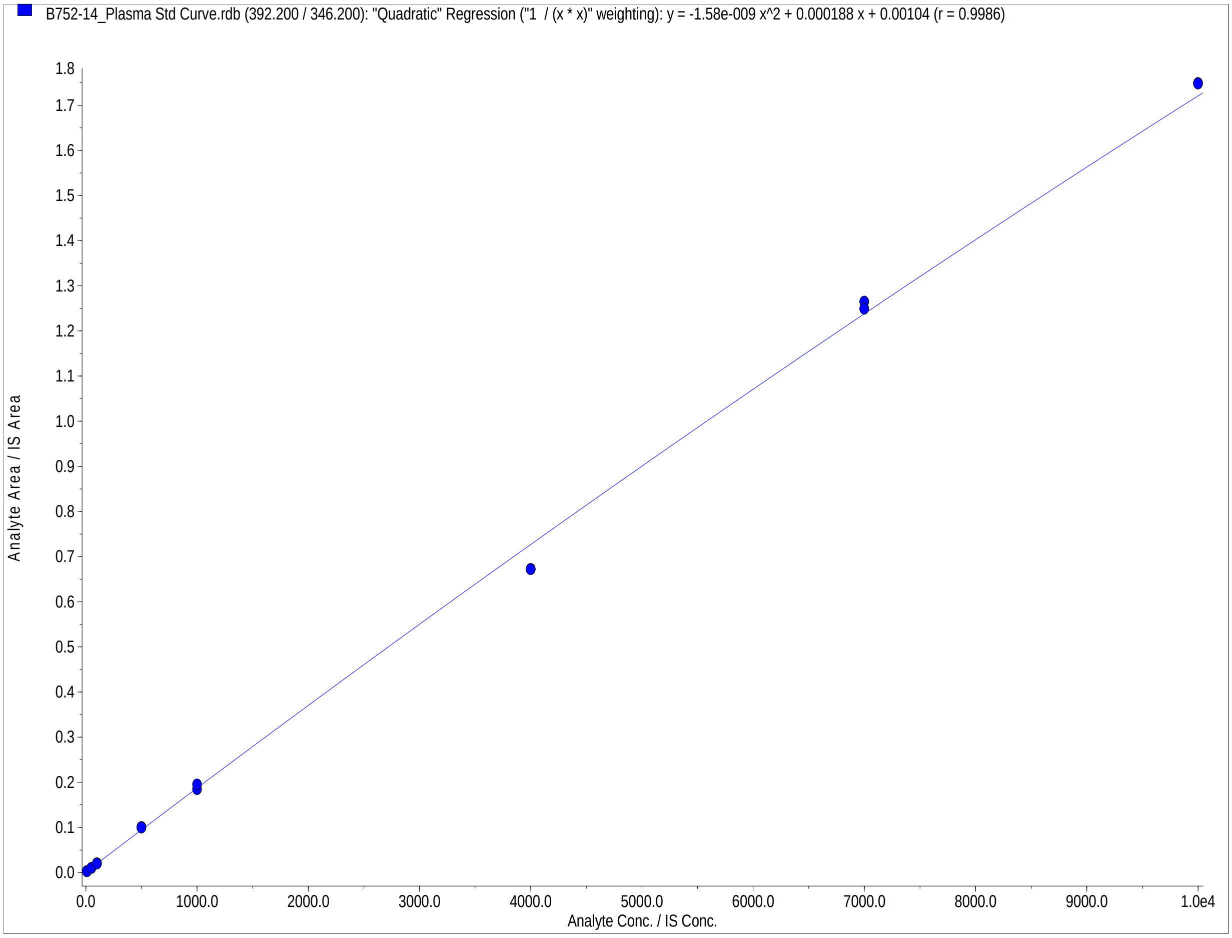

Figure B-1. Cynomolgus monkey plasma standard curve for analysis of BPN-14136

Table B-1
Accuracy of BPN-14136 Plasma QC Samples

Nominal and calculated concentrations shown are in ng/ml

QC Level<br>Nominal Conc. (ng/ml)	Low QC<br>20.0	Med. QC<br>4500	High QC<br>9500
n	4	4	4
Mean Conc.	18.4	4480	10425
SD	1.28	51.6	150
%CV	6.97	1.15	1.44
Mean % Accuracy	91.8	100	110

Pharmacokinetic Study of BPN-14136 Following a Single Dose Administration to Male Beagle Dogs

II. MATERIALS AND METHODS

A. Materials

Acetonitrile and formic acid were purchased from Mallinckrodt Baker, Inc. (Phillipsburg, NJ). Dimethyl sulfoxide (DMSO) was purchased from Sigma-Aldrich (St. Louis, MO). Milli-Q water was obtained in-house. Male Beagle dog plasma was purchased from Bioreclamation Inc. (Hicksville, NY). The analytical HPLC column was purchased from Phenomenex Inc. (Torrance, CA).

BPN-14136 and BPN-13671 (internal standard) were provided by the sponsor and the purity was assumed to be 100%.

B. Bioanalytical Method

Calibration Standards and QCs. Dog plasma calibration standards and quality control (QC) samples were prepared in untreated plasma as follows. A primary stock solution of 1 mg/ml analyte in DMSO was prepared, then dilutions performed to make spiking solutions of analyte in DMSO at various concentrations. One volume of spiking solution was added to 99 volumes of blank dog plasma to attain nominal concentrations of standards with a final non-plasma matrix concentration of 1.0%. The calibration standards and QCs were stored at -70°C until analysis, and duplicate aliquots of the standards used in the plasma sample analysis to generate the calibration curve, and n=6 replicates of the QCs per concentration used to monitor method accuracy.

The calibration standard and QC concentrations (ng/ml) in the plasma analyses were as follows: Standards: 10.0, 50.0, 100, 500, 1010, 4000, 7000 and 10000. QCs: 20.0, 4500, and 9500. Plasma blanks were also prepared and included in the sample analyses.

Plasma Extraction Method: Plasma standards/samples were stored frozen at -70°C and thawed to room temperature for approximately 20 minutes on the day of analysis. The extraction method was as follows: To 50 µl of plasma in a 2 ml microfuge tube was added 300 µl acetonitrile containing 500 ng/ml BPN-13671 (internal standard). Tubes were vortexed at maximum speed on a plate vortexer 5 minutes, centrifuged 8 minutes at approximately 18,000 g, and 40 µl of the supernatant removed to a glass

HPLC vial containing 1000 µl of 85/15/0.1: water/acetonitrile/formic acid (v/v/v). The vials were briefly vortexed to mix, prior to LC-MS/MS analysis.

LC-MS/MS Method: Samples were analyzed by LC-MS/MS using the following conditions:

LC Conditions

Instruments:	Shimadzu LC-20AD Pumps<br>Leap Technologies CTC HTS PAL Autosampler
Autosampler Temperature:	10°C
Column:	Phenomenex Luna C18(2) 50 x 2 mm, 5 µm
Column Temp.:	Room temperature
Mobile Phase:	A= 0.1% formic acid in water (v/v)<br>B= 0.1% formic acid in acetonitrile (v/v)
Elution Mode:	Gradient;

Time (min)	%A	%B	Flow rate (ml/min)
0.0	98	2	1.0
0.7	98	2	1.0
1.7	5	95	1.0
3.5	5	95	1.0
3.7	98	2	1.0
5.2	98	2	1.0

Compound	Retention Time (min)
BPN-14136	1.89
BPN-13671 (IS)	1.77

Injection Volume:	10 µl
Strong Needle Wash Solvent:	99/1: methanol/ammonium hydroxide (v/v)
Weak Needle Wash Solvent:	20/80/0.1: acetonitrile/water/formic acid (v/v/v)

MS Conditions

Instrument:	AB Sciex QTrap 5500
Ionization:	Electrospray ionization, positive ion mode
Detection:	Multiple Reaction Monitoring (MRM);

Analyte	MRM Transition (m/z)	Collision Energy (eV)	Declustering Potential
BPN-14136	392.2→346.2	33	70
BPN-13671 (IS)	389.2→159.1	60	110

Dwell Times:	75 msec
Resolution:	Unit mass resolution at Q1 and Q3
Curtain Gas:	32
Ion Spray Voltage:	4500V

Desolvation Temperature:	600°C
Ion Source Gas 1:	70
Ion Source Gas 2:	90
Entrance Potential:	6V (BPN-14136), 10V (BPN-13671)
Exit Potential:	18V (BPN-14136), 20V (BPN-13671)
Quantitation:	Integration and Quantitation by Analyst Software ver 1.4.2

Calibration Curves. Calibration standard curves were prepared as follows: the peak area of BPN-14136 was divided by the peak area of BPN-13671 (internal standard) to yield a peak area ratio (PAR). The resulting PARs vs. nominal concentrations for calibration standards were fitted to a 1/x weighted linear regression, to generate a calibration curve for quantitation of study samples.

These calculations were performed by the Analyst software. The goodness of fit of the standard curve is indicated by the coefficient of determination (r^2) obtained from the quadratic regression, with perfect fit yielding an r^2 value of 1.000.

Lower Limit of Quantitation (LLOQ). The LLOQ was defined as the lowest concentration that could be measured yielding back-calculated accuracy of at least 50% of the standards within 80-120% of nominal, and was set as the lowest concentration in the standard curve. In the plasma sample analyses the LLOQ was 10.0 ng/ml.

Linearity and Range: The range of the plasma standard calibration curve (LLOQ to upper limit of quantitation, ULOQ) was 10.0 to 10,000 ng/ml. The coefficient of determination (r^2) was ≥ 0.999 in the sample run. The calibration curve from the plasma sample run is shown in Figure C-1.

Accuracy and Precision of QC Samples: The accuracy (% of nominal) and precision (%CV) of the QC samples analyzed in the plasma sample analyses are shown in Table C-1.

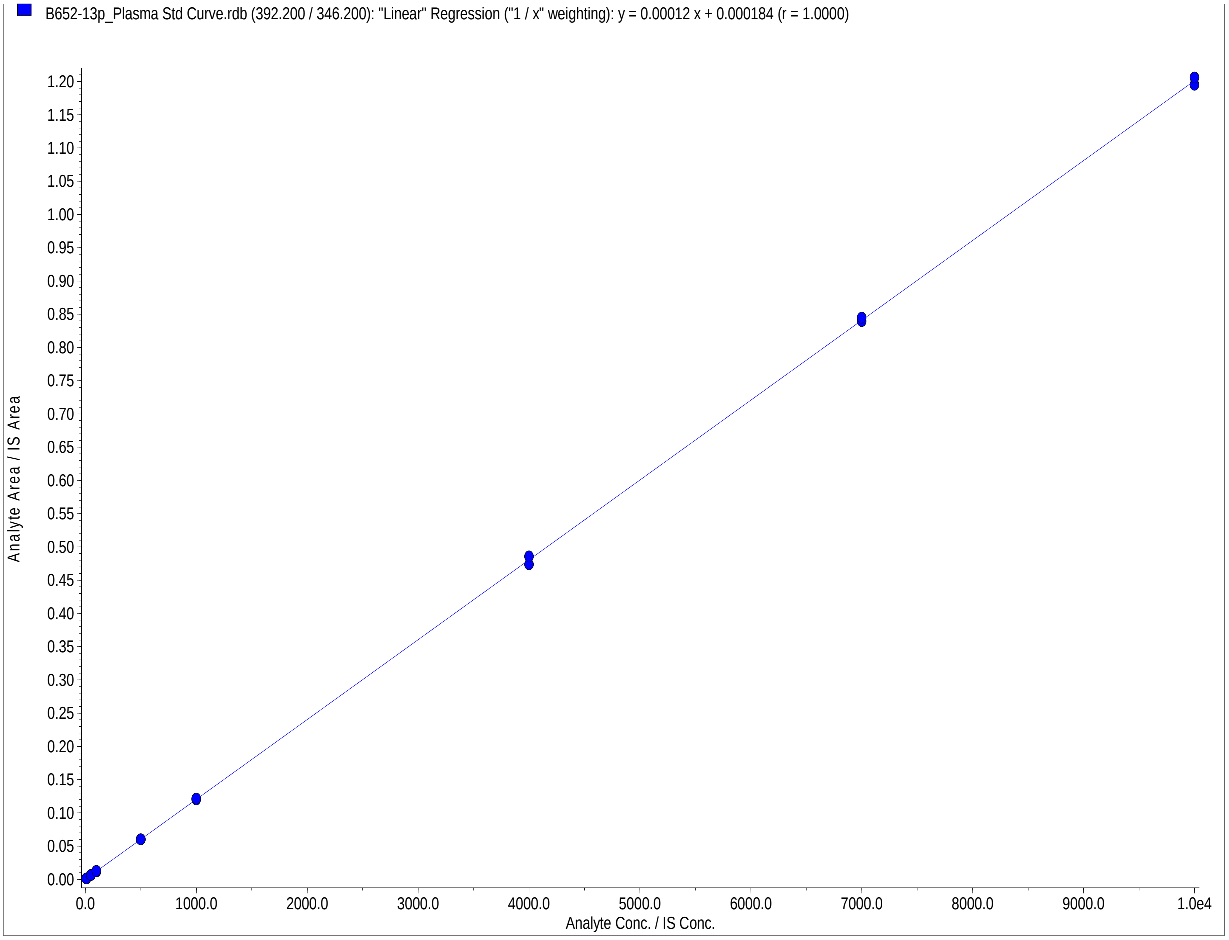

Figure C-1. Dog plasma standard curve for analysis of BPN-14136

Table C-1
Accuracy of BPN-14136 Plasma QC Samples

Nominal and calculated concentrations shown are in ng/ml

QC Level<br>Nominal Conc. (ng/ml)	Low QC<br>20.00	Med. QC<br>4500	High QC<br>9500
n	6	6	6
Mean Conc.	20.13	4490	10233
SD	2.06	59.3	137
%CV	10.2	1.32	1.34
Mean % Accuracy	101	100	108
