# Supplementary material for: Comparative pharmacokinetics and pharmacodynamics of the advanced Retinol-Binding Protein 4 antagonist in dog and cynomolgus monkey
Source: PLoS One. 2020 Jan 24;15(1):e0228291. doi: 10.1371/journal.pone.0228291 (PMC6980506; doi:10.1371/journal.pone.0228291)
Supplement: S2 File — (PDF) [file pone.0228291.s003.pdf]

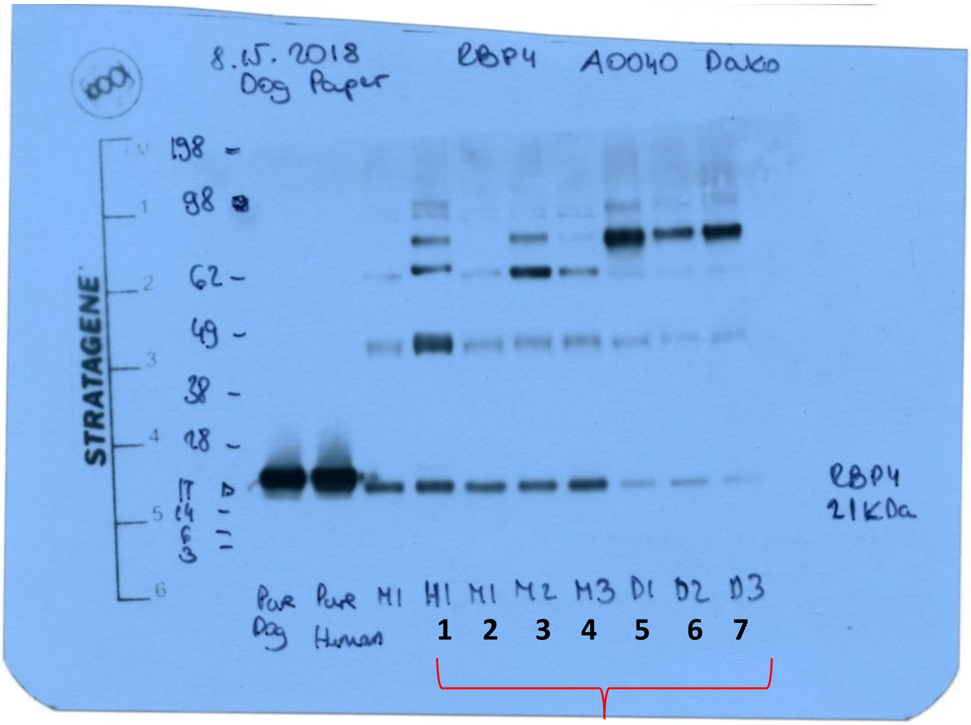

Figure 3, Upper Panel B,

Shorter exposure was performed for the lower portion of the same blot:

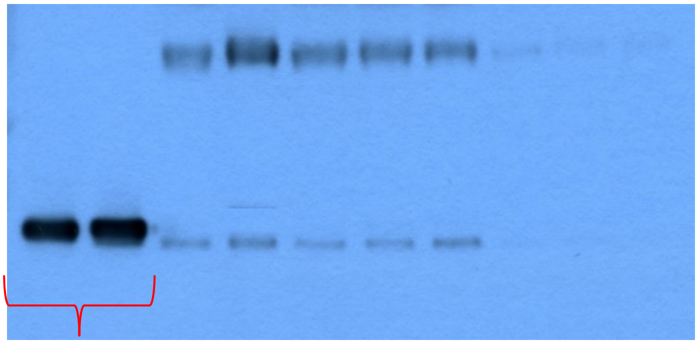

1 2

Figure 3, Panel A

Same blot was partially stripped and re-probed with the anti-transferrin antibody producing a 76 kDa band. The lower 21 kDa RBP4 bands can still be seen despite stripping:

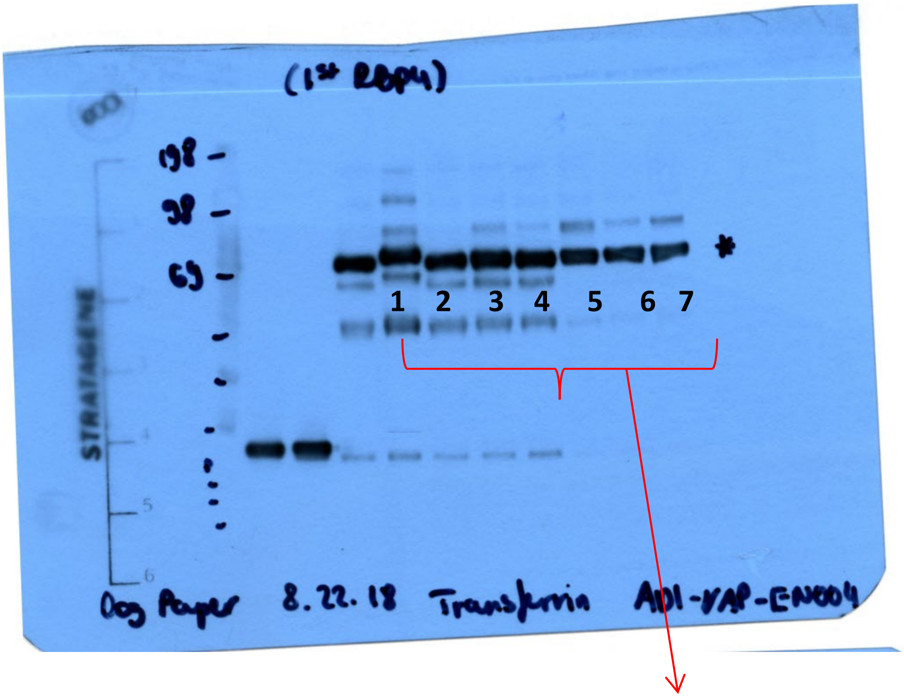

Figure 3, Lower Panel B,
